# New Anthropometric Measurements: Relationship to Thyroid Functions in Euthyroid Obese Subjects

**DOI:** 10.7759/cureus.20435

**Published:** 2021-12-15

**Authors:** Şevin Demir, Yasin Kara, Merve Melikoğlu, Kadriye Aydın, Ayşenur Özderya, Huriye Ecem Subaşı, Mustafa Reşat Dabak, Şule Temizkan

**Affiliations:** 1 Department of Family Medicine, Maltepe University Faculty of Medicine, Istanbul, TUR; 2 Department of Family Medicine, Şavşat Family Health Center No: 1, Artvin, TUR; 3 Department of Family Medicine, İstanbul Maltepe County Health Department, Istanbul, TUR; 4 Department of Endocrinology, Health Sciences University Kartal Dr Lütfi Kırdar Training and Research Hospital, Istanbul, TUR; 5 Department of Endocrinology, Acıbadem University Hospital, Istanbul, TUR; 6 Department of Family Medicine, Baskent University İstanbul Medical Research and Practice Center, Istanbul, TUR; 7 Department of Family Medicine, Health Sciences University İstanbul Haseki Training and Research Hospital, Istanbul, TUR; 8 Department of Endocrinology, Baskent University İstanbul Medical Research and Practice Center, Istanbul, TUR

**Keywords:** tsh, free t3, body round index, body adiposity index, a body shape index

## Abstract

Introduction

Body mass index (BMI) is unable to make a distinction between muscle mass and fat mass. Therefore, new anthropometric measurements, such as a body shape index (ABSI), body round index (BRI), and body adiposity index (BAI), have been formulated in recent years. Many studies have reported a correlation between BMI and thyroid function. In this study, we aimed to investigate the relationship between the above-mentioned new anthropometric measurements and thyroid functions in euthyroid obese subjects.

Methods

We included 675 euthyroid (TSH ≥ 0.4 and < 4.5 mIU/l) individuals from the obesity outpatient clinic, aged between 18 and 65 years old, with BMI ≥ 30. Thyroid-stimulating hormone (TSH), free T4 (fT4) and free T3 (fT3), anthropometric measurements (weight, height, and waist circumference), and bioelectric impedance analyses [percent body fat (PBF) and fat-free mass (FFM)] of individuals were measured and recorded. ABSI, BRI, and BAI were calculated with the data from these measurements. Anthropometric measurements were compared to thyroid function tests.

Results

Eighty percent of the subjects were female. The mean age and BMI were 38 ± 17 years and 38 ± 6 kg/m^2^, respectively. TSH was found to be negatively correlated with ABSI (p = 0.006) and positively correlated with BAI (p < 0.001), but a statistically significant relationship with BRI (p = 0.193) was not determined. Free T4 was not associated with any of the anthropometric measurements.While fT3 was determined to be positively correlated with ABSI (p = 0.008) and negatively correlated with PBF and BAI (p = 0.001, p = 0.002, respectively), no statistically significant relationship with fT3 and BRI was determined.

Conclusion

TSH is positively correlated with measurements of adiposity such as BMI, PBF, BAI while indexes in which abdominal obesity increases, such as waist circumference (WC), waist-hip ratio (WHR), and ABSI, are correlated with fT3 levels.

## Introduction

Obesity is a chronic disease characterized by excessive fat accumulation in the body, resulting from an imbalance of increased energy intake and reduced energy expenditure on the basis of an interaction of genetic and environmental factors [1].

Obesity and thyroid diseases are common in the general population. Thyroid hormones are important determinants of energy expenditure that also have effects on appetite. Many hormones, such as leptin, are secreted from fatty tissues and can alter the activity of the hypothalamus-pituitary-thyroid axis by influencing the central nervous system (CNS) to signal the amount of energy stored. Leptin levels rise with obesity, causing thyrotropin-releasing hormone (TRH) production in the paraventricular nucleus of the hypothalamus. As a result, TSH levels and thyroid hormone levels rise [2]. Leptin also alters the activity of deiodinases, thus promoting the conversion of T4 to T3 [3]. As a result of these interactions, the thyroid functions of obese individuals may vary depending on the adiposity distribution of the body.

Body fat content and distribution indexes are currently used as indicators of health and risk of cardiovascular diseases (CVDs), diabetes, and mortality. While body mass index (BMI) is considered a simple and useful method to diagnose obesity and can be used to predict associated consequences, there are concerns that BMI does not accurately reflect the amount and distribution of fat and muscle mass. Three new anthropometric metrics have recently been developed that take these aspects into account. In 2011, Bergman et al. described body adiposity index (BAI), in 2012, Krakauer et al. designed a body shape index (ABSI), and in 2013, Thomas et al. developed another new geometric calculation method, body roundness index (BRI) [4-6]. Several studies have reported a correlation between CVD, diabetes risk, and these new anthropometric indices [7-9].

While there are many studies evaluating the relationship between thyroid function tests and BMI, there are no studies examining this relationship with the previously mentioned new obesity indices. In this study, we investigated the relationship between BAI, ABSI, BRI, and thyroid function tests.

## Materials and methods

Subjects

We evaluated 675 euthyroid (TSH ≥ 0.4 and < 4.5 mIU/l) obese individuals (BMI ≥ 30 kg/m^2^) admitted to our obesity outpatient clinic between 2015 and 2017. Demographic characteristics, including weight, height, waist circumference, body composition analyses, comorbidities, and concomitant medications were obtained from patient files in the obesity outpatient clinic. Patients with chronic diseases (diabetes mellitus and thyroid, cardiovascular, pulmonary, and psychiatric diseases), as well as patients using drugs that would affect thyroid functions (i.e.; lithium, steroid, antiepileptics) were excluded.

The study was conducted in adherence to the Declaration of Helsinki II. The study protocol was approved by the local ethics committee (Kartal Dr. Lutfi Kirdar Training and Research Hospital Ethics Committee, Approval Date:11/01/2017, Approval Number: 2017/514/99/6).

Measurements and calculations

Bodyweight (kg) and height (m) were measured while subjects wore light clothing and no shoes. Waist circumference (WC) was measured at the umbilicus using non-elastic tape, at the end of a normal expiration in a standing posture. Hip circumference (HC) was measured at the level of maximum extension of the buttocks in the horizontal plane. The body mass index (BMI; kg/m^2^) was calculated as weight in kilograms divided by height in square meters. Bioelectrical impedance (Jawon GAIA 359 Plus, Body Composition Analyser, Kyungsan, Korea) was performed on all subjects at first clinic admission to measure fat-free mass (FFM) and percentage body fat mass (PBF). Other anthropometric indices were calculated from the following formulas: BAI = [hip circumference / height^3/2^ −18], ABSI = [WC / (BMI^2/3^ × height^1/2^)], BRI = [364.2 - (365.5 × √1 − (( WC / 2𝜋)^2^ / (0.5 × height)^2^)].

Fasting blood samples were collected in the morning following at least 12 hours of fasting. Plasma levels of TSH (mIU/l, reference range 0.4-4.5), fT4 (pmol/l, reference range 7.7-16), and fT3 (pmol/l, reference range 3.1-5.8), were determined by a chemiluminescence immune analyzer method (Beckman Coulter Inc., Fullerton, CA). Anti-thyroid peroxidase (TPO) and anti-thyroglobulin (anti-Tg) antibodies were measured by a chemiluminescence immunoanalysis method (Beckman Coulter Inc.). Anti-Tg antibodies had upper range values of 4.0 IU/ml while anti-TPO antibodies had upper range values of 9.0 IU/ml.

Statistical analyses

All statistical analyses were performed using Statistical Package for Social Sciences® software (SPSS Inc. Released 2008. SPSS Statistics for Windows, Version 17.0. Chicago: SPSS Inc.). The normality of data distribution was tested using the Kolmogorov-Smirnov test. For continuous variables, results are presented as mean ± standard deviation (SD) or median (25% and 75% interquartile) for non-normally distributed variables.

## Results

The general characteristics of patients are presented in Table 1. The average age was 38 ± 17 years and 82% of the study population was female. The average TSH level of patients was 2.0 ± 0.9 mIU/l. Eleven percent of the study population had anti-TPO positivity and 9% had anti-Tg positivity. The average BMI was 38 ± 6 kg/m^2^.

**Table 1:** Characteristics of the study population TPO: thyroid peroxidase; TSH: thyroid-stimulating hormone; WC: waist circumference; WHR: waist-hip ratio; BMI: body mass index; PBF: percentage body fat; FFM: fat-free mass; ABSI: a body shape index; BAI: body adiposity index; BRI: body roundness index

N	675
Age (year)	38±17
Female (%)	82
Thyroid tests	
TSH (mIU/l)	2.0±0.9
fT4 (pmol/l)	10.3±1.6
fT3 (pmol/l)	5.4±0.7
Anti-TPO positivity (%)	11
Anti-Tg positivity (%)	9
Anthropometric measurements	
Weight (kg)	99±18
WC (cm)	111±13
WHR	0.89±0.07
BMI (kg/m^2^)	38±6
PBF (%)	40±5
FFM (kg)	55±19
ABSI	0.0775±0.0054
BRI	7.83±2.12
BAI	43.2±7.6

Table 2 depicts the relationship between new and traditional anthropometric measurements. ABSI had a statistically significant positive correlation with WC and waist-hip ratio (WHR), as well as a statistically significant negative correlation with PBF and BMI, whereas BRI had a statistically significant positive correlation with WC, WHR, FFM, PBF, and BMI. BAI demonstrated a statistically significant positive correlation with WC, WHR, PBF, and BMI but a statistically significant negative correlation with FFM.

**Table 2:** The relation of new anthropometric measurements with classical measurements WC: waist circumference; WHR: waist-hip ratio; BMI: body mass index; PBF: percentage body fat; FFM: fat-free mass

	ABSI		BRI		BAI	
	r	p	r	p	r	p
Weight	-0.001	0.975	0.536	<0.001	0.201	<0.001
WC	0.506	<0.001	0.865	<0.001	0.335	<0.001
WHR	0.744	<0.001	0.392	<0.001	0.721	<0.001
FFM	0.092	0.017	0.125	0.001	-0.131	0.001
PBF	-0.271	<0.001	0.443	<0.001	0.721	<0.001
BMI	-0.206	<0.001	0.795	<0.001	0.731	<0.001

**Figure 1:**
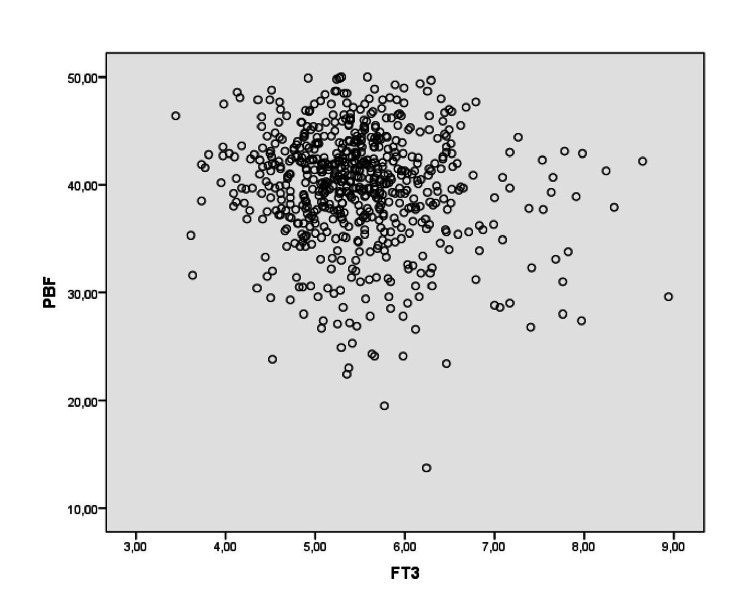
The relationship between fT3 and percent body fat

Table 3 shows the relationship between thyroid functions and anthropometric measurements. TSH had a negative correlation with ABSI (p = 0.006) and WHR (p = 0.017) and a positive correlation with BAI (p < 0.001), BMI (p = 0.002), and PBF (p < 0.001); however, it did not have a statistically significant correlation with BRI (p = 0.193). Free T4 was not associated with any of the anthropometric measurements. Free T3 was positively correlated with ABSI (p = 0.008), WC, and WHR (both p < 0.001) and negatively correlated with PBF and BAI (p = 0.001, p = 0.002, respectively) and had no significant relationship with BMI, FFM, or BRI. We also evaluated our findings with patients who are positive for antithyroid antibodies removed from the data set. No statistically significant difference was found between the correlations of patients with and without antibody positivity (Table 4). Figure 1 demonstrates the relationship between fT3 and PBF.

**Table 3:** Relationship between thyroid functions and anthropometric measurements WC: waist circumference; WHR: waist-hip ratio; BMI: body mass index; PBF: percentage body fat; FFM: fat-free mass; ABSI: a body shape index; BAI: body adiposity index; BRI: body roundness index

	TSH		fT4		fT3	
	r	p	r	p	r	p
Weight	0.060	0.118	0.016	0.692	0.151	<0.001
WC	0.018	0.647	0.010	0.808	0.141	<0.001
WHR	-0.093	0.017	-0.007	0.866	0.192	<0.001
BMI	0.122	0.002	0.006	0.880	0.029	0.459
PBF	0.174	<0.001	0.026	0.515	-0.127	0.001
FFM	0.021	0.591	-0.006	0.877	0.058	0.144
ABSI	-0.106	0.006	0.001	0.989	0.104	0.008
BRI	0.050	0.193	0.001	0.987	0.044	0.266
BAI	0.126	0.001	0.004	0.913	-0.123	0.002

**Table 4:** Relationship between thyroid functions and anthropometric measurements (patients with negative anti-thyroid antibodies) WC: waist circumference; WHR: waist-hip ratio; BMI: body mass index; PBF: percentage body fat; FFM: fat-free mass; ABSI: a body shape index; BAI: body adiposity index; BRI: body roundness index

	TSH		fT4		fT3	
	r	p	r	p	r	p
Weight	0.052	0.219	0.023	0.596	0.159	<0.001
WC	0.008	0.850	0.028	0.516	0.158	<0.001
WHR	-0.090	0.033	-0.006	0.890	0.170	<0.001
BMI	0.110	0.009	0.025	0.552	0.048	0.264
PBF	0.158	<0.001	0.047	0.269	-0.106	0.014
FFM	0.026	0.541	-0.009	0.830	0.046	0.290
ABSI	-0.104	0.013	0.006	0.892	0.110	0.011
BRI	0.076	0.201	0.037	0.543	0.080	0.188
BAI	0.111	0.008	0.030	0.485	-0.085	0.050

Patients were divided into two groups using a TSH cutoff of 2.5 (Table 5). A statistically significant difference in terms of BMI, PBF, WHR, BAI, and ABSI values was observed between these two groups of patients.

**Table 5:** Comparison of anthropometric measurements according to TSH quantiles WC: waist circumference; WHR: waist-hip ratio; BMI: body mass index; PBF: percentage body fat; FFM: fat-free mass; ABSI: a body shape index; BAI: body adiposity index; BRI: body roundness index; TSH: thyroid-stimulating hormone

	TSH<2.5 (n=477)	TSH≥2.5 (n=198)	p
fT3	5.5±0.8	5.4±0.6	0.090
fT4	10.6±1.6	9.9±1.9	<0.001
Weight	99±17	101±18	0.180
WC	111±13	112±13	0.440
BMI	38±6	39±7	0.002
PBF	39±5	41±5	<0.001
FFM	54±11	55±31	0.420
WHR	0.90±0.08	0.88±0.07	0.050
BRI	7.7 ±2.1	8.0±2.1	0.080
BAI	42.6±7.5	44.7±7.6	0.001
ABSI	0.0777±0.0055	0.0767±0.0051	0.040

## Discussion

It is known that hypothyroidism causes weight gain, and weight gain increases thyroid hormone levels through a variety of mechanisms. As the first trigger point of these mechanisms is not known, the results of adiposity in different body types are unclear. In this study, we attempted to understand this mechanism more clearly by comparing thyroid function tests with classical obesity criteria and new obesity indices such as ABSI, BRI, and BAI.

Bastemir et al compared the relationship between serum TSH levels and adiposity in a population of 226 euthyroid overweight or obese female individuals and 39 lean controls. Similar to our findings, the study reported that TSH levels were positively correlated with BMI and the degree of obesity while at the same time no correlation was found between serum fT4 and any of the parameters [10].

Bjergved et al tested the association between TSH and body weight in 1944 individuals followed for 11 years. They found a statistically significant positive association between changes in TSH concentrations and weight changes in both sexes. Weight increased by 0.6 kg in euthyroid women and 0.7 kg in euthyroid men for every one unit of TSH (mIU/l) increase. TSH change in women was classified into four quartiles, with the lowest quartile gaining 1.2 kg and the highest quartile gaining 3.6 kg. However, there was no link determined between baseline TSH concentration and weight gain, or between BMI and TSH change [11].

A decrease in fT3 and TSH levels after weight loss has been reported in both bariatric surgery and calorie-restriction studies [12-13]. In addition, a statistically significant positive correlation was shown between leptin levels and 24-hour TSH concentrations in calory restricted individuals [13]. Increased deiodinase activity leading to a high conversion rate of T4 to T3 for a defense mechanism to combat fat accumulation by boosting energy expenditure could be one possible reason for the presence of a link between obesity indices and fT3 in our study. Leptin also enhances the activity of deiodinases [14]. In a study by De Pergola et al., it was observed that both fT3 and the fT3/fT4 ratio were correlated with weight, BMI, and WC [15]. In our study, a positive correlation was observed between fT3 and weight and WC although no correlation was found with BMI.

A study by Liu G et al. was conducted with 569 obese euthyroid participants over a period of two years to observe the role of thyroid functions in diet-induced weight loss. They demonstrated that higher levels of baseline fT3 and fT4 but not TSH could predict weight loss. Also changes in free and total T3 levels, but not free and total T4 or TSH, were shown to be associated with changes in body weight. In their study, while fT3 was positively and fT4 was negatively associated with baseline BMI, TSH was not significantly associated [16].

In a study of 2524 euthyroid obese patients, Roef et al. determined that fT3 was positively associated with WC, BMI, whereas fT4 had a weak association. TSH was not found to be associated with BMI and WC [17].

There are several studies that have, as in our study, reported a positive correlation between TSH and BMI [10,12-13], and some studies that, in contrast to our findings, have reported no associations between TSH and BMI [16,18]. Free T3, in some studies, was found to be associated with BMI in contrast to our study, while some studies, like ours, determined no associations [18-19]. The cause of the different findings may be the different characteristics of the study participants or an underestimation of obesity. To our knowledge, the current study is among the first to examine the relationship between thyroid hormones and new obesity indices. We used new obesity indices in addition to traditional indices in order to measure obesity and the relationship between obesity and thyroid function more accurately.

ABSI considers waist circumference, body weight, and height as the main criteria. This index has been established in studies to be sufficient for determining the abdominal fat mass and superior to BMI and WC measurements for predicting premature death [5]. In the Women’s Health Initiative study, ABSI showed a linear association with mortality in postmenopausal women [20]. It has also been suggested that ABSI can be used to predict the occurrence of diabetes in individuals [21-22]. In 2013, Thomas et al. developed another new geometric calculation method: the BRI [6]. BRI values range from 1 to 16. Individuals with higher than this range have been shown to be at risk for CVD, but it was not superior to WC or BMI in this regard [23].

Eighty percent of patients in our study were women and it is known that females are more susceptible to gaining fat around the hips. As body fat percentage increases TSH and therefore fT3 and fT4 increase. An increase in abdominal obesity leads to a release in leptin that then leads to increased iodinase activity. With the increasing deiodinase activity, fT4 turns into fT3 and returns to its former level, and fT3 increases even more. Increasing fT3 decreases TSH levels with negative feedback. It is therefore that indexes in which body fat ratio increases, such as BMI, PBF, and BAI, are correlated with TSH while indexes in which abdominal obesity increases, such as WC, WHR, and ABSI, are correlated with fT3 levels.

Limitations

Obesity is more common in females compared to males, and it is more likely that females present to hospitals due to obesity. A vast majority of patients included in this study are females. While interpreting the results, the body structure of the female population was taken into consideration.

## Conclusions

Our study has demonstrated that TSH is positively correlated with BMI, PBF, and BAI while it is negatively correlated with WHR and ABSI. fT3 is positively correlated with weight, WC, WHR, and ABSI and negatively correlated with PBF and BAI.

Although there are many studies showing the relationship between thyroid functions and body mass index, to the best of our knowledge, there is no study showing the relationship between thyroid hormones and new obesity indices that can also show body fat distribution. We believe the correlations put forth by this study will shed light on our understanding of hormonal mechanisms.
